# Patient Attitudes Toward Mobile Phone-Based Health Monitoring: Questionnaire Study Among Kidney Transplant Recipients 

**DOI:** 10.2196/jmir.2284

**Published:** 2013-01-08

**Authors:** John William McGillicuddy, Ana Katherine Weiland, Ronja Maximiliane Frenzel, Martina Mueller, Brenda Marie Brunner-Jackson, David James Taber, Prabhakar Kalyanpur Baliga, Frank Anton Treiber

**Affiliations:** ^1^Division of Transplant SurgeryDepartment of SurgeryMedical University of South CarolinaCharleston, SCUnited States; ^2^College of NursingMedical University of South CarolinaCharleston, SCUnited States; ^3^College of MedicineMedical University of South CarolinaCharleston, SCUnited States

**Keywords:** cellular phone, attitude, kidney transplantation, telemedicine, mobile phone, smartphone, mhealth

## Abstract

**Background:**

Mobile phone based remote monitoring of medication adherence and physiological parameters has the potential of improving long-term graft outcomes in the recipients of kidney transplants. This technology is promising as it is relatively inexpensive, can include intuitive software and may offer the ability to conduct close patient monitoring in a non-intrusive manner. This includes the optimal management of comorbidities such as hypertension and diabetes. There is, however, a lack of data assessing the attitudes of renal transplant recipients toward this technology, especially among ethnic minorities.

**Objective:**

To assess the attitudes of renal transplant recipients toward mobile phone based remote monitoring and management of their medical regimen; and to identify demographic or clinical characteristics that impact on this attitude.

**Methods:**

After a 10 minute demonstration of a prototype mobile phone based monitoring system, a 10 item questionnaire regarding attitude toward remote monitoring and the technology was administered to the participants, along with the 10 item Perceived Stress Scale and the 7 item Morisky Medication Adherence Scale.

**Results:**

Between February and April 2012, a total of 99 renal transplant recipients were identified and agreed to participate in the survey. The results of the survey indicate that while 90% (87/97) of respondents own a mobile phone, only 7% (7/98) had any prior knowledge of mobile phone based remote monitoring. Despite this, the majority of respondents, 79% (78/99), reported a positive attitude toward the use of a prototype system if it came at no cost to themselves. Blacks were more likely than whites to own smartphones (43.1%, 28/65 vs 20.6%, 7/34; *P*=.03) and held a more positive attitude toward free use of the prototype system than whites (4.25±0.88 vs 3.76±1.07; *P*=.02).

**Conclusions:**

The data demonstrates that kidney transplant recipients have a positive overall attitude toward mobile phone based health technology (mHealth). Additionally, the data demonstrates that most kidney transplant recipients own and are comfortable using mobile phones and that many of these patients already own and use smart mobile phones. The respondents felt that mHealth offers an opportunity for improved self-efficacy and improved provider driven medical management. Respondents were comfortable with the idea of being monitored using mobile technology and are confident that their privacy can be protected. The small subset of kidney transplant recipients who are less interested in mHealth may be less technologically adept as reflected by their lower mobile phone ownership rates. As a whole, kidney transplant recipients are receptive to the technology and believe in its utility.

## Introduction

Nearly 400,000 people living in the United States suffer with end stage renal disease, of these; approximately 93,000 are awaiting kidney transplantation [1,2]. Kidney transplantation is the preferred mode of treatment for end stage renal disease as it offers superior quality of life and improved life expectancy compared to chronic dialysis [3-6]. Outcomes after kidney transplantation are negatively impacted by poor medication adherence and suboptimal control of common comorbid medical conditions such as hypertension and diabetes [7-10]. Black patients suffer disproportionately with end stage renal disease and represent the vast majority of patients on dialysis in South Carolina [1]. Black kidney transplant recipients suffer a poorer graft survival than white kidney transplant recipients [11-17]. The reasons for this may include poorer medication adherence [11,18], heightened immunological response [19,20] and a higher prevalence of comorbid illnesses [1,21]. The development of effective, efficient and non-intrusive approaches to aid kidney transplant recipients self-management and monitoring is critical to success as limited healthcare provider resources are increasingly taxed by growing demand.

Recent studies have suggested that remote monitoring via mobile health technology (mHealth) is an effective and sustainable strategy for facilitating patient provider communication, improving health outcomes, increasing adherence to medical regimens and reducing costs in some chronic illnesses [22-30]. Mobile phone based monitoring is an attractive option due to their ubiquity, connectivity, computational power, portability and relatively low cost [23,25,31-32]. A critical component to the success of any mHealth system is the willingness and ability of the target population to adopt and effectively utilize the technology. Previous studies have investigated the attitudes of different patient populations in regards to mobile phone based remote monitoring in other disease states [33-36]. Kidney transplant recipients are a unique population due to the high complexity of their medical regimens, the critical importance of strict medication adherence, the near universal presence of significant comorbid medical illness and the geographic distance that often separates them from their transplant center [7,9,37-38]. Kidney transplant recipients also tend to be relatively aged, a factor that may lead this population to be less willing or able to successfully utilize advanced technologies such as smart mobile phones or Bluetooth enabled medical devices.

The aims of this study were twofold. First, to assess the attitudes of a racially diverse sample of kidney transplant recipients on the use of a mHealth remote monitoring system, particularly to enhance medication adherence and blood pressure control. Second, to investigate the whether demographics, prior technology utilization, stress levels and self-reported medication adherence impact on the attitudes toward mHealth.

## Methods

### Participants and Recruitment

Study participants were recruited from the Kidney Transplant Clinic at the Medical University of South Carolina (MUSC), Charleston. Eligible patients were those who had previously received a kidney transplant, were over 18 years of age and spoke English. Between February and April 2012, 103 patients were approached during their usual post-transplant clinic visit, either by their clinical coordinator, clinic nurse or physician. Patients were asked if they were willing to speak to the study coordinator regarding participating in the survey, 99 agreed to participate. Those that declined did so either for lack of time or lack of interest. The demographic and transplant related clinical characteristics of the survey participants are summarized in Table 1. The study was approved by the MUSC institutional review board.

### Study Setting and Design

Patients were approached about completing a survey that included questions on their attitudes toward remote monitoring, mobile phones, electronic medication monitors and electronic home blood pressure monitors. Also evaluated were perceived levels of general stress and medication adherence (Table 1). Patients were individually shown to a private clinic space, accompanied by their informal caregivers (if present), where they were provided a description and demonstration of a prototype mHealth system with a presentation of the specific steps that were required to utilize the device (Multimedia Appendix 1).

The prototype system included a smartphone (Motorola Droid X), a wireless (GSM-enabled) medication tray (Maya MedMinder) and a wireless (Bluetooth-enabled) blood pressure monitor (Fora D15b). Patients were required to use the medication tray for all medication dispensing and to measure their resting blood pressure and pulse every third day at both morning and evening. The smartphone automatically transfers the blood pressure and pulse data to computer servers for later analysis. The medication tray is fully programmable and capable of delivering reminders in the form of light, tone, text message or phone call. Adherence is tracked in real time and can trigger the delivery of motivational or positive reinforcement messages to the patient via text, email or phone. A summary of the adherence over time can be generated and delivered via email to the patient and the treating clinician. Blood pressure and pulse readings outside predetermined safe parameters could generate automated alert messages that would be sent to both patient and physician. Participants were informed that a clinical coordinator would contact them in the event that alerts were generated.

### Medication Adherence Scale

Medication adherence was evaluated using a 7 item modified Morisky Medication Adherence Scale with an internal consistency of 0.82 and a sensitivity and specificity of 91% and 50% respectively [39]. Further description and psychometric data on the Morisky scale are described in detail elsewhere [40]. The modified Morisky scale yields a score in the range of 0 to 7 with higher scores reflecting higher adherence to medication. Scores can be categorized into high (=7), moderate (≥6 but <7) and poor (<6) adherence levels based on its criterion validity with blood pressure control among hypertensive patients [39].

### Perceived Stress Scale

Generalized perceived stress was measured using the Perceived Stress Scale in which each of the 10 items is answered using a 5 point Likert item ranging from ‘never’ to ‘very often’. The scale has established psychometric properties including internal consistency of 0.85 [41]. Internal consistency for the present study was 0.85.

### mHealth Related Survey

The respondents’ awareness of, and attitudes toward, mHealth and telemedicine based remote monitoring were evaluated using a 10 item survey (Table 2), in which 9 of the 10 items were answered using a 5 point Likert item ranging from ‘strongly disagree’ to ‘strongly agree’. The items were adapted from prior studies assessing patients attitudes toward mobile phone based remote monitoring for chronic illness [34,36,42]. Items included questions on their perceptions of remote monitoring and their comfort with using mobile phones. Cronbach’s alpha internal consistency coefficient was 0.92 for the 9 items. The 10^th^ item queried their *a priori* awareness of health related remote monitoring technology.

Our sample was analyzed in two ways: by race, blacks versus whites (Table 3) and by attitude toward use of mHealth. Participants who answered either ‘agree’ or ‘strongly agree’ to the question whether they would use the mHealth system if it were free were compared to the participants who chose either ‘disagree’, ‘strongly disagree’ or ‘neutral’ (Table 4). Means and standard deviations for continuous variables and frequency distributions for categorical variables were used to describe the characteristics of the total sample and the racial and mHealth attitude groups. Clinical and demographic features were compared for racial and attitude groups using the pooled t test for continuous variables and the X^2^ test/Fisher exact test for categorical variables.

## Results

### Demographic and Clinical Characteristics

The results indicate that 85% (83/98) of respondents are presently using devices at home to monitor either their blood pressure or blood sugar. Nearly two thirds of the respondents, 63% (62/99), were using medication dispensing devices (ie. standard non-signaling pillboxes). Respondents reported slightly lower than average levels of perceived stress (Table 1) that were not remarkably higher than the general population (10.0 ± 6.5 out of 40) [43]. Self-reported adherence was also reported to be moderately high (6.1 out of 7) [39]. There were no racial differences in perceived stress (*P*=.13), but there was a difference in self-reported adherence (*P*=.03).

### Mobile Phone Utilization

Nearly all respondents (90%, 87/97) indicated that they already own a mobile phone and were comfortable utilizing this technology. Over a third of the patients (35%, 35/99) reported that they own a smartphone and over half (52%, 51/99) reported that they had access to a working smartphone in the household other than their own. Most of the patients (82%, 81/99), indicated that there was someone in the household who could assist them with using a mobile phone if needed.

The survey results also indicated that most respondents had a familiarity with using mobile phones for reasons other than phone calls. Amongst these, over half (61%, 60/99) used a mobile phone to send or receive text messages, 38% (38/99) to browse the web, 35% (35/99) to send or receive email and 34% (34/ 99) reported using a mobile phone to download a ringtone or a mobile application.

**Table 1:** Demographic and transplant related clinical characteristics of survey participants.

Variable	Mean (± SD) or Proportion
Age	53.1 ± 13.4 (Median 52)
Gender (Male)	65% (64/98)
Race (Black )	66% (65/99)
Marital status (Married)	64% (63/98)
Education level (≤High School )	38% (38/99)
Employment (Part or Full Time)	22% (21/98)
Annual income (<$30,000)	57% (44/77)
Months since kidney transplant	29.2 ± 54.5
More than one transplant	12% (12/99)
Perceived Stress Scale score	10.9 ± 6.5
Morisky Scale score	6.1 ± 1.1

### Attitudes and Willingness to Use mHealth Technology

Only 7% (7/98) of participants had any prior knowledge of mHealth remote monitoring technology before being surveyed (Table 2). However, most of the participants felt that mobile technology would be helpful in reminding them to follow their doctor’s directions (81%, 80/99). The majority also felt that the technology would allow their doctor to make more rapid adjustments to their medication regimen (84%, 83/99). Furthermore, most of the participants, 79% (78/99), indicated that if they were provided the mHealth system at no cost and instructed on its use that they would use it as directed by their health care provider. The addition of free technical support did not significantly increase their willingness to use the devices. On the matter of health information, 80% (79/99) indicated that they were comfortable with a health care provider monitoring their health information using remote monitoring technology and 76% (75/99) felt confident that their privacy could be adequately protected. Almost all participants (95%, 94/99) felt that it was important to follow their doctor’s directions and 87% (86/99) thought that remote monitoring technology would help them effectively communicate with their health care providers about their medical conditions. When asked about how they would prefer to receive instructions from their health care providers, most respondents preferred that communication be done via phone call, with voicemail being the most common second choice. Text messaging was the third most common choice, with only a small fraction of patients indicating that they were interested in receiving instruction primarily via live video conferencing.

**Table 2:** Responses to mHealth related survey.

Survey Items	Mean (± SD) or Percentage
Heard of tele-health (yes)	7% (7/98)
Would use mHealth devices if free	4.08 ± 0.98
If someone available to answer questions likely to use devices as directed	4.18 ± 0.92
Comfortable having health monitored remotely by doctor/nurses using mHealth technologies	4.16 ± 0.89
Comfortable using cell phone	4.30 ± 0.80
Mobile technology will help remind me to follow doctor’s directions	4.14 ± 0.89
Mobile technology will allow doctor to make medication changes quicker	4.22 ± 0.79
Confident privacy protected when using mHealth devices	4.07 ± 0.92
Important to follow doctor’s directions	4.56 ± 0.72
Confident mHealth technology can effectively communicate my medical condition to my doctor	4.24 ± 0.81

### Racial Differences

As shown in Table 3, when compared to whites, blacks were younger (*P*=.04), more likely to report hypertension as an etiology of their renal failure (*P*=.05), more likely to be a first time recipient of a kidney transplant (*P*=.02) and more likely to live nearer to the transplant center (*P*<.001). While mobile phone ownership did not differ significantly between races, blacks were more likely than whites to own a smartphone (*P*=.03). Although it did not reach statistical significance, there was a trend toward more mobile phone based internet usage among blacks (*P*=.08). Perceived stress levels did not vary by race (*P*=.13), but there was a significant difference in medication adherence with blacks reporting slightly poorer adherence (*P*=.03). Blacks had a slightly more positive attitude toward mHealth than whites as gauged by their willingness to use the technology if it came at no cost to them (*P*=.02). There were no significant differences between blacks and whites in the level of education (*P*=.62), annual income (*P*=.16) or employment status (*P*=.46).

**Table 3:** Race comparison on transplant related characteristics, stress exposure, medication adherence and cell phone ownership.

		Black	White	Degrees of Freedom	*P*
Age		51 ± 13.1	56.9 ±13.4	96	.04
Gender (Male)		65%	65%	1	.99
Primary cause for kidney failure				6	.05
	HTN^a^ (alone or + other)	39%	21%		
	Diabetes (alone or + other)	22%	18%		
	Diabetes + HTN^a^	15%	9%		
	Other	20%	35%		
	Not sure	5%	18%		
More than one transplant (yes)		6%	24%	1	.02
Travel time to transplant center (< 2 hours)		77%	33%	3	<.001
Own mobile phone		86%	97%	1	.16
Own smartphone		43%	21%	1	.03
Would use mHealth devices if free		4.3 ± .88	3.8 ± 1.1	3	.02
Perceived Stress Scale score		11.7 ± 6.8	9.5 ± 5.7	97	.13
Morisky Scale score		5.9 ± 1.2	6.5 ± 0.9	97	.03

^a^ Hypertension

### Characteristics of Respondents with Positive Attitude Toward mHealth

Respondents who answered either ‘agree’ or ’strongly agree’ to the query as to whether they would use the mHealth system, as demonstrated, if it were free, were more likely to be employed (*P*=.04), the recipient of their first transplant (*P*=.02), already using a medication tray at home (*P*=.04) and the owner of a working mobile phone (*P*=.04). These respondents were also more likely to own a smartphone (*P*=.01) and to have used a mobile phone to text (*P*=.02), email (*P*=.01), browse the internet (*P*=.002) or download an application or ringtone (*P*=.03). As can be seen in Table 4, they also reported higher levels of perceived stress (*P*=.01).

**Table 4:** Comparisons of patients who do versus do not favor use of mHealth devices.

		Agree	Do Not Agree	Degrees of Freedom	*P*
Age		52 ± 13.1 (Median 51)	57 ± 14.3 (Median 62)	96	.13
Race				1	.01
	Black	86%	14%		
	White	65%	35%		
Socioeconomic status					
	Employment (Part or Full Time)	27%	5%	1	.04
	Education level (≤High School)	37%	43%	2	.89
	Annual income (<$30,000)	45%	43%	1	.54
More than one kidney transplant		8%	29%	1	.02
Use medication dispensing device at home		68%	43%	1	.04
Own cell phone		93%	76%	1	.04
Own smartphone		42%	10%	1	.005
Perceived Stress Scale score		11.7 ± 6.5	7.7 ± 5.4	97	.01
Morisky Scale score		6.0 ± 1.2	6.6 ± 0.7	97	.04

## Discussion

Recent literature demonstrates that mHealth technology can have a positive impact on the quality of life, self-efficacy and the ability to monitor biochemical or physiologic markers of disease control across a wide array of illnesses [44]. While the evidence is mixed as to the cost effectiveness of mHealth technology at present [45], it seems reasonable to hypothesize that it will become cost effective, as the demand increases, cost of the technology decreases and the long-term health benefits are realized. Furthermore, as penetrance of the smartphone technology increases amongst consumers, it seems likely that there will be an increasing demand for this type of health care delivery from patients.

### Principal Results

With 90% (87/97) of respondents owning a mobile phone and 35% (35/99) owning a smartphone, this population of kidney transplant recipients closely mirrors the adult American population [46]. This finding is mildly surprising given South Carolina’s historically low household income and underscores the near ubiquitous use of mobile phone technology. Interestingly, in our cohort, while there were no racial differences in overall mobile phone ownership, blacks were significantly more likely to own a smartphone. This reflects national figures that show higher rates of smartphone adoption among racial minority groups [47]. Blacks are significantly more likely than whites to use a wireless device to access the internet [46]. As early adopters and high utilizers, blacks may be uniquely positioned to benefit from improving mHealth technology. The penetrance of mobile phone technology, particularly the rapid ascension of the smartphone [47], bodes well for the continued expansion of the mobile phone’s role in health care delivery.

Few of the kidney transplant recipients had any knowledge of mHealth technology prior to being surveyed. Despite that, the vast majority was receptive to utilizing such technology if the devices were provided at no cost. Respondents felt that having the technology would help them follow their medical regimen and improve communication with their healthcare providers, particularly with regards to the efficiency of regulating or changing their medical regime as the need arises. These findings are consistent with other studies that have evaluated the attitudes toward mHealth technology among patients with various chronic illnesses, including essential hypertension, diabetes and congestive heart failure [27,34,36].

Although there was a high receptivity toward using mHealth technology, there was a cohort of respondents who indicated a less than positive attitude. That these respondents were less likely to own a mobile phone and far less likely to own a smartphone might reflect a lower level of comfort with technology. This potential barrier to use of an mHealth system could be addressed both by making the system easier to use and by providing some skilled assistance and training. The fact that these same respondents were more likely to have had a prior kidney transplant and were less likely to be using a medication tray at home, might indicate a higher comfort level with immunosuppression medications or a lower perceived importance of medication adherence. That this cohort self-reports higher adherence with medications and lower levels of perceived stress may, in part, explain their diminished interest in the technology. Unsurprisingly, this same cohort of patients was less likely to respond positively to the remaining questions regarding comfort with being monitored, comfort with mobile phone technology, privacy protection and various aspects of utility.

### Limitations

These findings must be evaluated within the context of several limitations of the study. First, that all respondents were recruited from a single transplant center, which may call into question the generalizability of the findings. However, it should be noted that this center is the sole transplant service provider for the State of South Carolina and has a catchment population of over 4.6 million persons. Second, those that chose to participate might be predisposed to a positive attitude toward mHealth and thereby introduce a positive bias. The participation of nearly everyone who was approached however, suggests that a significant bias toward mHealth is unlikely. Third, it cannot be assumed that the respondents’ purported interest in mHealth will translate into actual use. Anecdotally, as we have begun to enroll kidney transplant recipients in a mHealth medication adherence trial, as a proof of concept research based on this work, we have experienced high participation and utilization rates.

### Conclusions

This is the first study assessing the attitudes of transplant recipients with this technology and the data demonstrates that there is a positive overall attitude towards mHealth technology. Additionally, the data demonstrates that most kidney transplant recipients already own and are comfortable using mobile phones and that many of these participants already own and use smart mobile phones. Results indicate that the participants feel that mHealth offers an opportunity for improved self-efficacy and improved provider driven medical management. Participants are also comfortable with the idea of being monitored using mobile technology and are confident that their privacy can be protected. As a whole, kidney transplant recipients are receptive to the technology and believe in its utility. Further research in this area should include patient centered evaluations of usability and usefulness as well as proof of concept trials to identify areas of concern.

## Multimedia Appendix 1

Patient demo video.

## Abbreviations

HTN: hypertension
mHealth: mobile phone based health technology

## References

[1] (2011). U.S. Renal Data System.

[2] (2011). OPTN: Organ Procurement and Transplant Network.

[3] Manninen DL, Evans RW, Dugan MK (1991). Work disability, functional limitations, and the health status of kidney transplantation recipients posttransplant. Clin Transpl.

[4] Laupacis A, Keown P, Pus N, Krueger H, Ferguson B, Wong C, Muirhead N (1996). A study of the quality of life and cost-utility of renal transplantation. Kidney Int.

[5] Wolfe RA, Ashby VB, Milford EL, Ojo AO, Ettenger RE, Agodoa LY, Held PJ, Port FK (1999). Comparison of mortality in all patients on dialysis, patients on dialysis awaiting transplantation, and recipients of a first cadaveric transplant. N Engl J Med.

[6] Neipp M, Karavul B, Jackobs S, Meyer zu Vilsendorf A, Richter N, Becker T, Schwarz A, Klempnauer J (2006). Quality of life in adult transplant recipients more than 15 years after kidney transplantation. Transplantation.

[7] De Geest S, Borgermans L, Gemoets H, Abraham I, Vlaminck H, Evers G, Vanrenterghem Y (1995). Incidence, determinants, and consequences of subclinical noncompliance with immunosuppressive therapy in renal transplant recipients. Transplantation.

[8] Nevins TE, Kruse L, Skeans MA, Thomas W (2001). The natural history of azathioprine compliance after renal transplantation. Kidney Int.

[9] Vlaminck H, Maes B, Evers G, Verbeke G, Lerut E, Van Damme B, Vanrenterghem Y (2004). Prospective study on late consequences of subclinical non-compliance with immunosuppressive therapy in renal transplant patients. Am J Transplant.

[10] Desmyttere A, Dobbels F, Cleemput I, De Geest S (2005). Noncompliance with immunosuppressive regimen in organ transplantation: is it worth worrying about?. Acta Gastroenterol Belg.

[11] Butkus DE, Meydrech EF, Raju SS (1992). Racial differences in the survival of cadaveric renal allografts. Overriding effects of HLA matching and socioeconomic factors. N Engl J Med.

[12] Fan PY, Ashby VB, Fuller DS, Boulware LE, Kao A, Norman SP, Randall HB, Young C, Kalbfleisch JD, Leichtman AB (2010). Access and outcomes among minority transplant patients, 1999-2008, with a focus on determinants of kidney graft survival. Am J Transplant.

[13] Chakkera HA, O'Hare AM, Johansen KL, Hynes D, Stroupe K, Colin PM, Chertow GM (2005). Influence of race on kidney transplant outcomes within and outside the Department of Veterans Affairs. J Am Soc Nephrol.

[14] Foster CE, Philosophe B, Schweitzer EJ, Colonna JO, Farney AC, Jarrell B, Anderson L, Bartlett ST (2002). A decade of experience with renal transplantation in African-Americans. Ann Surg.

[15] Eckhoff DE, Young CJ, Gaston RS, Fineman SW, Deierhoi MH, Foushee MT, Brown RN, Diethelm AG (2007). Racial disparities in renal allograft survival: a public health issue?. J Am Coll Surg.

[16] Opelz G, Mickey MR, Terasaki PI (1977). Influence of race on kidney transplant survival. Transplant Proc.

[17] Rostand SG (1992). US minority groups and end-stage renal disease: a disproportionate share. Am J Kidney Dis.

[18] Weng FL, Israni AK, Joffe MM, Hoy T, Gaughan CA, Newman M, Abrams JD, Kamoun M, Rosas SE, Mange KC, Strom BL, Brayman KL, Feldman HI (2005). Race and electronically measured adherence to immunosuppressive medications after deceased donor renal transplantation. J Am Soc Nephrol.

[19] Kerman RH, Kimball PM, Van Buren CT, Lewis RM, Kahan BD (1991). Stronger immune responsiveness of blacks vs whites may account for renal allograft survival differences. Transplant Proc.

[20] Leffell MS, Steinberg AG, Bias WB, Machan CH, Zachary AA (1994). The distribution of HLA antigens and phenotypes among donors and patients in the UNOS registry. Transplantation.

[21] Rostand SG, Brown G, Kirk KA, Rutsky EA, Dustan HP (1989). Renal insufficiency in treated essential hypertension. N Engl J Med.

[22] Krishna S, Boren SA (2008). Diabetes self-management care via cell phone: a systematic review. J Diabetes Sci Technol.

[23] Logan AG, McIsaac WJ, Tisler A, Irvine MJ, Saunders A, Dunai A, Rizo CA, Feig DS, Hamill M, Trudel M, Cafazzo JA (2007). Mobile phone-based remote patient monitoring system for management of hypertension in diabetic patients. Am J Hypertens.

[24] Scherr D, Kastner P, Kollmann A, Hallas A, Auer J, Krappinger H, Schuchlenz H, Stark G, Grander W, Jakl G, Schreier G, Fruhwald FM (2009). Effect of home-based telemonitoring using mobile phone technology on the outcome of heart failure patients after an episode of acute decompensation: randomized controlled trial. J Med Internet Res.

[25] Krishna S, Boren SA, Balas EA (2009). Healthcare via cell phones: a systematic review. Telemed J E Health.

[26] Pare G, Sicotte C, St-Jules D, Gauthier R (2006). Cost-minimization analysis of a telehomecare program for patients with chronic obstructive pulmonary disease. Telemed J E Health.

[27] Quinn CC, Clough SS, Minor JM, Lender D, Okafor MC, Gruber-Baldini A (2008). WellDoc mobile diabetes management randomized controlled trial: change in clinical and behavioral outcomes and patient and physician satisfaction. Diabetes technology & therapeutics.

[28] Kauer SD, Reif SC, Crooke AH, Khor A, Hearps SJ, Jorm AF, Sanci L, Patton G (2012). Self-monitoring using mobile phones I the early stages of adolescent depression: randomized controlled trial. J Med Internet Res.

[29] Vuong AM, Huber JCJr, Bolin JN, Ory MG, Moudini DM, Helduser J, Begaye D, Bonner TJ, Forjuoh SN (2012). Factors affecting acceptibility and usability of technological approaches to diabetes self-management: a case study. Diabetes Technol Ther.

[30] Seto E, Leonard KJ, Cafazzo JA, Barnsley J, Masino C, Ross HJ (2012). Mobile phone-based telemonitoring for heart failure management: a randomized controlled trial. J Med Internet Res.

[31] Boland P (2007). The emerging role of cell phone technology in ambulatory care. J Ambul Care Manage.

[32] Seto E, Leonard KJ, Cafazzo JA, Barnsley J, Masino C, Ross HJ (2012). Perceptions and experiences of heart failure patients and clinicians on the use of mobile phone-based telemonitoring. J Med Internet Res.

[33] Proudfoot J, Parker G, Pavlovic DH, Manicavasagar V, Adler E, Whitton A (2010). Community attitudes to the appropriation of mobile phones for monitoring and manageing depression, anxiety, and stress. J Med Internet Res.

[34] Bostock Y, Hanley J, McGown D, Pinnock H, Padfield P, McKinstry B (2009). The acceptability to patients and professionals of remote blood pressure monitoring using mobile phones. Prim Health Care Res Dev.

[35] Cleland J, Caldow J, Ryan D (2007). A qualitative study of the attitudes of patients and staff to the use of mobile phone technology for recording and gathering asthma data. J Telemed Telecare.

[36] Seto E, Leonard KJ, Masino C, Cafazzo JA, Barnsley J, Ross HJ (2010). Attitudes of heart failure patients and health care providers towards mobile phone-based remote monitoring. J Med Internet Res.

[37] Axelrod DA, Dzebisashvili N, Schnitzler MA, Salvalaggio PR, Segev DL, Gentry SE, Tuttle-Newhall J, Lentine KL (2010). The interplay of socioeconomic status, distance to center, and interdonor service area travel on kidney transplant access and outcomes. Clin J Am Soc Nephrol.

[38] Axelrod DA, Guidinger MK, Finlayson S, Schaubel DE, Goodman DC, Chobanian M, Merion RM (2008). Rates of solid-organ wait-listing, transplantation, and survival among residents of rural and urban areas. JAMA.

[39] Bharmal M, Payne K, Atkinson MJ, Desrosiers MP, Morisky DE, Gemmen E (2009). Validation of an abbreviated Treatment Satisfaction Questionnaire for Medication (TSQM-9) among patients on antihypertensive medications. Health Qual Life Outcomes.

[40] Morisky DE, Ang A, Krousel-Wood M, Ward HJ (2008). Predictive validity of a medication adherence measure in an outpatient setting. J Clin Hypertens (Greenwich).

[41] Cohen S, Kamarck TMermelstein R (1983). A global measure of perceived stress. J Health Soc Behav.

[42] Pinnock H, Slack R, Pagliari C, Price D, Sheikh A (2006). Professional and patient attitudes to using mobile phone technology to monitor asthma: questionnaire survey. Prim Care Respir J.

[43] Spacapan S, Oskamp S (1988). Perceived stress in a probability sample of the United States. The social psychology of health.

[44] Pare G, Moqadem K, Pineau G, St-Hilaire C (2010). Clinical effects of home telemonitoring in the context of diabetes, asthma, heart failure and hypertension: a systematic review. J Med Internet Res.

[45] Mistry H (2012). Systematic review of studies of the cost-effectiveness of telemedicine and telecare. Changes in the economic evidence over twenty years. J Telemed Telecare.

[46] Smith A (2011). Americans and their cell phones. Pew Internet & American Life Project.

[47] (2012). Nielsen Wire.

